# A survey on HIV-related health-seeking behaviors among transgender individuals in Jakarta, based on the theory of planned behavior

**DOI:** 10.1186/s12889-015-2480-0

**Published:** 2015-11-16

**Authors:** Ciptasari Prabawanti, Arie Dijkstra, Pandu Riono, Gagan Hartana

**Affiliations:** Social Psychology Department, Faculty of Behavioral and Social Sciences, University of Groningen, Grote Kruisstraat 2/1 9712 TS, Groningen, The Netherlands; Population Studies and Biostatistic Department, Faculty of Public Health, University of Indonesia, Kampus UI, Depok, 16424 West Java Indonesia; Faculty of Psychology, University of Indonesia, Kampus UI, Depok, 16424 West Java Indonesia

**Keywords:** Jakarta-Indonesia, Transgender, Health-seeking behavior, STI services, HIV test, Theory of planned behavior

## Abstract

**Background:**

Male-to-female transgender (*waria*) individuals are at high risk for HIV. This study aims at mapping the psychological determinants of four HIV-related health-seeking behaviors. This knowledge can be used to develop effective interventions to prevent the spread of HIV/AIDS.

**Methods:**

The study involved 209 *waria* from five districts in Jakarta, selected with a cluster sampling procedure. Cross-sectional data were gathered through structured interviews. The four examined behaviors are, visiting sexually transmitted infections (STIs) services regularly, adherence to STI treatment, taking an HIV test and picking up the result of HIV test. For all four behaviors, specific measures of the psychological determinants as defined by the Theory of Planned Behavior were developed: attitudes, subjective norms and perceived behavioral control (PBC). Logistic regression analyses were conducted with these three psychological measures as independent variables and the behaviors as dependent variables.

**Results:**

Of the 209 *waria,* 20.6 % had never visited STI services in the last 6 months, while 56.5 % had visited the services once or twice, and 23 % had visited the service three or more times. A HIV test had been taken by 90.4 % of the *waria*, and of those, 64.6 % had picked up the results. About 85 % of the *waria* who did a HIV test had been tested for HIV one or two times in the last 6 months and 10 % had been tested three to four times. The variance in behaviors that was explained by the concepts defined in the Theory of Planned Behavior ranged from 15 to 70 %; PBC was the most powerful predictor. Furthermore, the results showed that in several cases the relationships of attitudes or subjective norms with the dependent variable were mediated by one or both other independent variables.

**Conclusions:**

The results regarding the prominent role of PBC suggest that interventions should increase *waria*’s control over the behavior: Engaging in specific desired behaviors should be made easier for them. Besides, *waria*’s attitudes and subjective norms should be addressed, by education, but possibly also by providing *waria* with a positive experience with the behavior, for example, by designing a professional and friendly health care system.

## Background

Male-to-female transgender (*wari*a) individuals are among the key affected populations in Indonesia, vulnerable to HIV and other sexually transmitted infections (STIs) because they often practice unprotected anal sex [1–3]. The Integrated Biological and Behavioral Survey (IBBS) among key affected populations in 2011 reported a prevalence of syphilis of 31 % (from 25.2 % in 2004) and of rectal gonorrhea and/or chlamydia of 38 % (from 42.1 % in 2004) in this group. The overall HIV prevalence among *waria* in five cities (Jakarta, Surabaya, Bandung, Semarang and Malang) in Indonesia was 22 %; it was the highest (31 %) in Jakarta [4, 5], while HIV prevalence in the general population in Indonesia is 0.33 % [6]. In addition, as previous studies indicated, an increase in STI may result in an increased risk of HIV transmission [3, 7]. For this reason, it is essential to integrate STI control programs into HIV risk reduction strategies.

Since the emergence of HIV in the 1980s, STI control efforts have been increasingly defined in relation to HIV program priorities. STI services have been integrated with HIV test as a package of services. This package aims to provide rapid and effective treatment to patients – either symptomatic or asymptomatic – to break the chain of infection and shorten the duration of infectivity. HIV test aims to detect HIV-positive people as early as possible, in order to avoid missed opportunities of accessing appropriate treatment and prevent transmission as a result of unknown HIV infection status. The package – STI services and HIV tests – has been selected as the first gate to access HIV-related services. The World Health Organization (WHO) stated that early HIV testing and counseling of STI patients, detection of acute HIV infection, and regular STI screening and treatment are promising interventions [8].

As shown in the latest IBBS 2011, the prevalence of HIV among *waria* in Jakarta is above 30 % [4]. Thus, it would be appropriate to aim to decrease HIV transmission by targeting *waria* as one of the key affected populations: The most cost-effective way of controlling the spread of STIs is exposing key affected populations to effective interventions. That is, the prevention efforts that effectively reduce transmission in the “core” population - whose members usually have many partners - are important and often sufficient to reduce transmission in the population at large [8]. For this reason, it is important to examine *waria’s* behaviors with regard to HIV-related health-seeking behaviors. In this study, we address four relevant behaviors.

The first relevant behavior is visiting STI services regularly. This means that *waria* commit themselves to visiting an STI clinic on a regular basis as part of an STI screening program. The guideline released by the Ministry of Health of the Republic of Indonesia (MOH RI) in 2011 recommends that the key affected populations, including people who frequently practice unprotected sexual behaviors, should visit STI services at least four times a year or on a monthly basis for the best results [9]. The second relevant behavior is taking an HIV test. *Waria* are strongly encouraged to take an HIV test during the *window period,* which means at least once in every 3–6 months [10]. The third relevant behavior is picking up the test result. That is, after having completed HIV test, patients should pick up the result, enabling them to access follow-up services such as a post-test counseling and necessary treatment. The fourth relevant behavior is treatment adherence. The patients who have attended STI services and have been diagnosed with STI should adhere to the medical prescription regarding the dose and duration of use. However, in many cases the patients do not adhere to the STI treatment [11].

The effectiveness of screening and treatments regarding HIV largely depend on *waria*’s behavior. When prevention interventions would accomplish to influence *waria*’s behavior in the desired direction, this would very probably reduce the spread of HIV. To be able to develop effective prevention interventions, it is important to have insight into the psychological determinants of the above four health-related behaviors.

One of the most recognized theories to understand health behaviors is the theory of planned behavior (TPB). The TPB postulates three conceptually independent determinants behavior. The first is the attitude toward the behavior; the degree to which a person has a favorable or unfavorable evaluation or appraisal of the behavior in question. The second determinant is psycho-social factor termed subjective norms; the perceived social pressure to perform or not to perform the behavior. The third determinant is the degree of perceived behavioral control (PBC); the perceived ease or difficulty of performing the behavior. The TPB predicts that the more favorable the attitude and subjective norms with respect to a behavior are, and the greater the PBC is, the stronger an individual’s intention to perform the behavior, and the higher the chance that the actual behavior will be performed [12]. In a review of the application of TPB to health-related behaviors, the TPB was able to explain a substantial proportion of variance in intention and future behavior, although the efficiency of the theory varied between health-related behavior categories: The average explained variance in intention was 40.9 %, varying from 32 % (eating behavior) to 46.8 % (oral hygiene behaviors). The average explained variance in behavior was 34 %, varying from 15.6 % (clinical and screening behaviors) to 42.3 % (HIV/AIDS-related behaviors) [13].

Nevertheless, there is a gap in the literature regarding understanding of people’s health-seeking behaviors in relation to STI. One of the few studies we found was an exploratory study applying TPB on stigma, STI and attendance to a Genito-Urinary Medicine (GUM) clinic in a close-knit community in the north of England [14]. Furthermore, a meta-analytic study on predicting intentions and attendance at screening programs using the Theory of Reasoned Action (TRA) identified 33 studies (e.g., cervical smear, genetic test, colorectal screen, mammogram, diabetes, TB screening, prenatal screening and health check) [15]. Thus, there are studies reported on health-seeking behavior that apply TPB, but very few are on STI screening and none on STI screening among *waria* [13–16].

With regard to behaviors related specifically to HIV testing, a few previous studies showed the applicability of the TPB. One study addressed the intended use of voluntary HIV test services [16]. The analyses indicated that the PBC and attitude towards using HIV test services were significant predictors of intention to use HIV test services, explaining 30 % of variance in the behavior. Another study reported psychological factors in association with the uptake of HIV tests among men who have sex with men in Hongkong [17]. This study indicated that the TPB variables were significantly associated with both lifetime and 12-month uptake of HIV tests.

Although all three determinants in the TPB have been found to be related to behavior, the TPB variables show differences in the proportions of the variance in specific behaviors they explain. For example, subjective norm was not related to several behaviors [18]. This would mean that interventions do not have to target subjective norms to influence behavior. However, an alternative explanation is that subjective norms have an indirect relation with the behavior; it might influence behavior *through* its influence on attitude and PBC. In such a case of mediation, interventions should also target subjective norms. Therefore, in the present study, we will test for possible mediation effects.

The first aim of this study was to examine the relationships of the TPB variables (i.e., attitude, subjective norms, PBC) with HIV-related health-seeking behaviors (visiting STI services regularly, adherence to STI treatment, taking an HIV test and picking up the result of the HIV test). On the basis of the TPB we hypothesized that specific measures of attitude, subjective norms and PBC would be related to each of the four health-seeking behaviors.

The second aim of this study was, when possible, to conduct mediation tests to examine whether subjective norms and PBC mediate the relationships of attitude with behavior, and whether attitude and PBC mediate the relationships of subjective norms with behavior. By increasing insight into the determinants of these behaviors, we hope to contribute to the development of effective interventions to fight the spread of HIV.

## Methods

### Recruitment procedure

We used maps from the transgender organization in Jakarta (Yayasan Srikandi Sejati) to select the sites of the study. The *waria* in this study were recruited using a cluster sampling procedure with the five municipalities in Jakarta as groups (Central, North, South, East and West), representing all residential locations of *waria*. In total, we recruited 210 participants from the Center of Jakarta (*n* = 23), East Jakarta (*n* = 75), West Jakarta (*n* = 45), South Jakarta (*n* = 45), and North Jakarta (*n* = 22) between September and October 2007. To gather information, interviews were conducted during the day, starting about 12 pm (in the afternoon) to 6–7 pm (in the evening). This seemed the best time for conducting interviews in *waria*, as they were still sleeping in the morning and they were starting to dress up and go out to the park, railway station and other places to meet the clients. The inclusion criteria were the following: Being identified as a transgender by mamis, living in one of the five selected districts in Jakarta, be able to speak and understand Bahasa Indonesia, and participate voluntarily in the study. One participant from East Jakarta was excluded from this study due to a serious language barrier. Therefore 209 *waria* participated, comprising almost 16 % of the last total estimated number of *waria* in Jakarta [19].

*Waria* usually migrate from rural areas to big cities like Jakarta. Upon arrival they will join and link with the *waria* community, that is coordinated by a senior ‘mami’ who provides guidance and protection. A mami usually provides support to the 5–15 *waria* in her group, including violence protection and promotion of condom use, uptake of HIV testing and regular STI check-ups [3]. ‘Mami’, as a senior person also will ensure that the newcomer will be socially accepted in the network. This support and protection are needed because *waria* are a stigmatized and discriminated group, who are easily recognized by their looks. *Waria* communities may provide *waria* with a “safe haven” of support and recognition.

Since mamis’ role may be rather instrumental, some of them are recruited and placed as field coordinators in the *waria* community organization. Therefore, a field coordinator is usually a key person in a district. By recruiting and cooperating with the mamis, the *waria* organization is able to provide the list of *waria*’s living areas and all mamis in the five districts were asked to contact participants for the assessments. In the selected areas, mami listed the *waria* who were present and available in sampled living areas at the time of data collection. All these *waria* were approached and included in the study.

The cooperation between mamis in five districts with the *waria* organization brings mutual benefits: The *waria* organization usually has easier access to the health services and social welfare programs which are managed by the district health and social welfare office, while mamis have ability to enter *waria’s* social networks and mobilize them.

*Waria* who live in the areas that were selected for this study, by design, have been exposed to HIV/AIDS infection prevention intervention and have access to HIV-related health services. The services were designed to cover the whole districts and all *waria* communities in Jakarta. Accordingly, we assume that all *waria* in Jakarta have been exposed to the intervention in some way. Therefore, to the extent that the present sample of *waria* is representative of the whole *waria* population in Jakarta, the results can be generalized to the Jakarta population. However, because the Jakarta population has been exposed by designed preventive interventions, care must be taken to generalize the present results to *waria* populations in different health care environments.

### Interview procedure

The study presented in this article was part of a broader study that covered condom use and HIV-related health-seeking behaviors. The ethical approval has been provided by the Ethical Committee Psychology University of Groningen in the Netherlands, and the IRB approval from the local institution was provided by the Ethical Committee of Psychology University of Indonesia. This article only provides data on health-seeking behaviors, including adherence to STI treatment. The study of condom use-related behaviors is presented in another paper [20].

The interviews were structured and conducted face-to-face. The interview first, assessed demographic variables, sexual history and sexual practices. Secondly, all the questions related to health-seeking behavior were asked: visiting STI services regularly, adherence to STI treatment, taking for HIV test and picking up the test result. The interviews took 45–60 min.

Due to *waria’s* limited understanding of the meaning of questions related to sexual and HIV-related health-seeking behaviors, this study selected the interview as a method to gather the data. The interviews had been conducted by using Bahasa Indonesia combined with local and slang language. Another consideration is that the illiteracy level among *waria* still is very high. For that reasons, the four interviewers were trained in advance by the first author to cope with limited literacy. Five interviewers, including the first author, were involved in the data collection.

Informed consent was prepared on two levels. First, permission was granted by the “mamis” as the coordinators and leaders of *waria* in each district. The permission was given after the first author conducted a meeting with all “mamis” from the five districts, to explain the purpose of the study and the requirement to have an interview only on voluntary basis. Secondly, the interviewers introduced themselves to the respondents and provided them with information about the purpose of the study. The interviewers explained the respondents that the participation was on a voluntary basis, meaning that they could withdraw at any time without having to state a reason. Subsequently, the respondents were asked whether they had understood the information and were willing to participate. The actual interview started after the individual had given a verbal consent to participate. When the respondents were under 18 years old, a verbal consent was given by the “mami” as the responsible person in each group. Unwritten informed consent was chosen for this study to assure the anonymity and confidentiality of the respondents.

The interviews were conducted at different venues. *Waria* usually live in groups, in the middle of a *kampong* (Spelled *kampung* in Malay and Indonesian). In Malaysia, Brunei, Indonesia and Singapore, the term kampong (village) applies to traditional villages, especially of indigenous people and the term has also been used to refer to urban slum areas and enclosed developments within towns and cities (https://en.wikipedia.org/wiki/Kampong), which is often quite crowded, and mixed with the general population. Through networks with *mamis*, we were able to find quiet places or separate rooms to conduct the interviews. The majority of the interviews were conducted in the *waria’s* bed rooms, a smaller number in the salons, and the remaining in the Primary Health Center (PHC) or the *mami’*s living rooms.

### Questionnaire

#### Background characteristics

The socio-demographics gathered were age, educational level, cultural background, persons currently living with the respondent, type of job and duration of stay in Jakarta. Sexual behavior was assessed with questions on commercial sex practices, current sex partners (only men/mostly men/men and women equally/mostly women/only women), the type of sex they most often engaged in (anal sex as the receptive partner/insertive partner/both as receptive and insertive partner/receiving oral sex/giving oral sex/insertive vaginal sex and others), condom use as the receptive partner in the past week, and condom use as the insertive partner in the past month.

The central part of the questionnaire was based on the application of the TPB on the four behaviors. The four examined behaviors are, visiting Sexually Transmitted Infection (STI) services regularly, adherence to the STI treatment, taking an HIV test and picking up the result of HIV test.

#### Attitude

The attitudes were assessed by using a 7-point semantic differential scale: *unhealthy* (1) – *healthy* (7); *harmful* (1) – *beneficial* (7); *boring* (1) – *exciting* (7); *inconvenient* (1) - *fun/enjoyable* (7); *something that can be overlooked* (1) - *something I have to do* (7); *foolish* (1) – *wise* (7). The items for each behavior category include the following: An example item for visiting STI services regularly: “If I go to STI services once every 2 or 3 months, this is…”; An example item for adherence to STI treatment: “If I receive drugs or get the prescription from the physician, finishing all drugs accordingly is…”; An example item for taking an HIV test: “If I take an HIV test at least once a year, this is…”; An example item for picking up the result of HIV test:,“If I pick up the results, this is…”. Concerning each behavior separately, the average item score was computed to be used as the scale score: The higher the score, the more positive the attitude towards the specific behavior. The Cronbach’s alphas of the four scales ranged from .70 to .88.

#### Subjective norms

The subjective norms were measured by using a 7-point semantic differential scale ranging from *should not* (1) to *should* (7). Items were related to normative beliefs regarding regular partner, friends, *mamis* and outreach workers. The items for each behavior category were the following: “According to the following people, I should or should not visit STI services once every 2 or 3 months”, “According to the following people, I should or should not finish all drugs received from or prescribed by the physician”, “According to the following people, I should or should not take an HIV test at least once a year”, “According to the following people, I should or should not pick up the results of HIV test (after taking an HIV test). Concerning each behavior separately, the average item score was computed to be used as the scale score: The higher the score, the more positive the subjective norms towards the specific behavior. The Cronbach’s alphas of the four scales ranged from .86 to .90.

#### Perceived behavioral control

PBC was measured by using a 7-point semantic differential scale. The first scale ranged from *very difficult* (1), *difficult* (2), *somewhat difficult* (3), *not easy* (4), *somewhat easy* (5), *easy* (6), to *very easy* (7). The second scale ranged from *not sure at all to be able* (1) to *very sure to be able* (7), and the last one was *completely uncertain to be able to ensure* (1) to *will certainly be able to ensure* (7). Each behavior was assessed with three items. Example items consisted of two questions and one statement: “In the next 6 months, how easy or difficult will it be for you to visit STI services?”, “In the next 6 months, if I want to, I am sure that I will be able to finish all drugs received from or prescribed by the physician”, “In the next 6 months, how sure are you that you will be able to take an HIV test?”. Concerning each behavior separately, the average item score was computed to be used as the scale score: The higher the score, the easier engaging in the specific behavior was perceived to be. The Cronbach’s alphas of the four scales ranged from .79 to .87.

#### Actual behavior

Each of the four behaviors was assessed with one item. They were: (1) “In the last 6 months, how many times have you visited STI services?” The responses indicated the number of times visiting STI services in the last 6 months; (2) “When you received medicine or got a prescription from the doctor, did you take or buy and finish it all?” The possible responses were *never* (1), *seldom* (2), *sometimes* (3), *regularly* (4), *often* (5), *very often* (6) and *always* (7); (3) “Did you take an HIV test?” (Yes/No); and (4) “Did you pick up the result of HIV test?” (Yes/No).

### Data analyses

Because two of the four behaviors were assessed with a binary variable, we decided to use logistic regression analysis for all four behaviors. Therefore, visiting STI services and adherence to STI treatment was also dichotomized (0 versus more than 0, and not always versus always, respectively). The covariates chosen were age, education and main job. The first analysis examined the relationships of these covariates with each of the DVs. Only when a covariate had a significant relationship with the DVs it was included in the main test: The three IVs – attitude, subjective norms and PBC – predicting each of the DVs. When one or more covariates were included, we present the statistics of the *improvement* of the statistical model by the set of three main predictors. When no covariate was included, we only present the statistics of the three main predictors.

To test for mediation effects, Preacher and Hayes’ SPSS Macro PROCESS (version 17) was used. Because this is a fairly novel method, it will be presented here in some detail. PROCESS has been described by Preacher and Hayes (2008) as a method for testing multiple mediators. This procedure yields unstandardized path coefficients for a multiple mediator model and estimates 95 % confidence intervals (CI) of the indirect (= mediated) effect using a bootstrapping sample procedure. Assessing an indirect effect through a bootstrapping sample procedure is more reliable than testing significance of the mediation effect, because the sampling distribution of the indirect effects is normal only for large samples. The mediation analysis used here followed the product of coefficients approach, and thus focused on the indirect effects rather than the individual paths. Using PROCESS for conducting the mediation test does not require normality in the selected variables [21].

The Mediate test was used here to examine whether 1) the relationships of subjective norms (IV) with behavior (DVs) were mediated by attitude and PBC, and whether; 2) the relationships of attitude (IV) with behavior (DVs) were mediated by subjective norms and PBC. The results below indicate mediation when the Confidence Interval of the indirect path by a 95 % bias corrected bootstrap (based on 5000 bootstrap samples) does not contain zero.

**Fig. 1:**
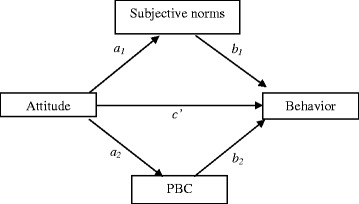
Indirect effects of attitude on behavior through subjective norms and perceived behavioral control

**Fig. 2:**
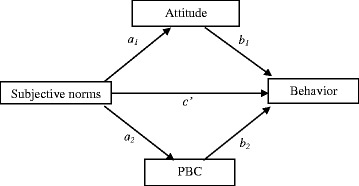
Indirect effects of subjective norms on behavior through attitude and perceived behavioral control

Since the model has more than one mediator, it is called a single-step multiple mediator model (see Figs. 1 and 2), in which the total effect of the model (the relationships of attitude or subjective norms with behaviors are symbolized by *c*) is equal to the direct effect of attitude or subjective norms on behavior which are symbolized by *c*_*’*;_ plus the sum of the indirect effect of attitude on behaviors through subjective norms (*a*_*1*_*b*_*1*_) or *PBC (a*_*2*_*b*_*2*_*)*, or plus the sum of the indirect effect of subjective norms on behaviors through attitude (*a*_*1*_*b*_*1*_) or PBC *(a*_*2*_*b*_*2*_*)*. That is, *c* = *c’* + *a*_*1*_*b*_*1*_ + *a*_*2*_*b*_*2.*_

## Results

### Background characteristics

In total, 209 *waria* took part in the study. The participants’ mean age was 30 years (range 15–67); 23 % had been in primary school, 28 % had been in school level 7–9, and almost 42 % had a higher education level, while 5 % had studied at a university. Most *waria* were unmarried (89.5 %), 5 % were married, and 5.8 % were divorced and separated.

Sex work is common among waria in Jakarta. Selling sex was an important source of income for *waria*: 35.9 % Reported selling sex as their primary source of income, while 24.4 % reported selling sex as their additional income. Thus, approximately 60 % of the 209 *waria* in our sample engaged in commercial sex.

Of the 209 *waria*, 20.6 % had never visited STI services, 56.5 % had visited the services once or twice, and 23 % three or more times, in the last 6 months. HIV test had been taken by 90.4 % (189) of the *waria*, and of those, 64.6 % had picked up the results. About 85 % of those who had had a test, had been tested for HIV one to two times and 10 % had been tested for HIV three to four times, in the last 6 months.

### Predicting behaviors

#### Visiting STI services regularly

Because data on the use of STI services of 3 participants were missing, the analyses were conducted in a sample of 206 *waria*. Only “main job” was included as a covariate. The results showed (in Table 1) that by adding the set of TPB predictors, the model significantly improved (chi-square = 21.79, *p* < .001, and *df* = 3), with an increase in Nagelkerke’s *R2* of .20. One predictor was significantly related to behavior: PBC (ExpB = 1.24), indicating that the higher the PBC, the higher the chance that *waria* scored high on behavior.

**Table 1:** Logistic regression analyses of behavior of HIV-related health-seeking behaviors

No	Variable	Behavior				
		Exp (β)	95 % CI		Nagelkerke R2	*N*
			Lower	Upper		
1	Visiting STI services regularly				.199	206
	Attitude	1.01	0.92	1.10		
	Subjective norms	0.10	0.91	1.10		
	Perceived behavioral control	1.24***	1.10	1.39		
2	Adherence to complete STI treatment				.699	67
	Attitude	1.14	0.82	1.57		
	Subjective norms	1.53	0.87	2.71		
	Perceived behavioral control	2.19**	1.36	3.53		
3	Taking an HIV test				.189	204
	Attitude	0.95	0.85	1.05		
	Subjective norms	1.10	0.97	1.25		
	Perceived behavioral control	1.19**	1.06	1.34		
4	Picking up the result of HIV test				.147	188
	Attitude	0.96	.88	1.06		
	Subjective norms	0.96	.87	1.06		
	Perceived behavioral control	1.27***	1.11	1.44		

*p < .05

**p < .01

***p < .001

#### Adherence to STI treatment

Adherence to STI treatment could only be analyzed in the 67 *waria* who had received an STI treatment. Only level of education was included as a covariate. The results showed (in Table 1) that by adding the set of TPB predictors, the model was significantly improved (chi-square = 39.34, *p* < .001, and *df* = 3), with an increase in Nagelkerke’s *R2* of .70. One predictor was significantly related to behavior: PBC (ExpB = 2.19), indicating that the higher the PBC, the higher the chance that *waria* scored high on behavior.

#### Taking for HIV test

Because data on taking for HIV test of 5 participants were missing, the analyses were conducted in a sample of 204 *waria*. Again, only level of education was included as a covariate. The results showed (in Table 1) that by adding the set of TPB predictors, the model significantly improved (chi-square = 14.20, *p* < .005, and *df* = 3), with an increase in Nagelkerke’s *R2* of .19. One predictor was significantly related to behavior: PBC (ExpB = 1.19), indicating that the higher the PBC, the higher the chance that *waria* scored high on behavior.

#### Picking up the results of HIV test

Because only 188 participants had had an HIV test, picking up the results of the test was only analyzed among these 188 *waria*. No covariate has to be included. The set of three TPB predictors showed (in Table 1), only one predictor had a significant relation with behavior (chi-square = 21.30, *p* > .001, and *df* = 3) with Nagelkerke’s *R2* of .15. PBC was significantly related to behavior (ExpB = 1.27), indicating that the higher the PBC, the higher the chance that *waria* scored high on behavior.

### Mediation testing for attitude and subjective norms

The findings from the logistic regression analyses showed that attitudes and subjective norms were not related to all four behaviors. This finding may have resulted from the relationship of attitudes with behavior being mediated by subjective norms and PBC (Fig. 1), and the relationship of subjective norms with behavior being mediated by attitude and PBC (Fig. 2). Therefore, we conducted mediation tests to examine these relations more closely, using the SPSS Macro PROCESS (version 17) by Preacher and Hayes [21]. Below (and in Tables 2 and 3) we present, firstly, the results concerning the mediation of attitude related to behavior; and secondly, the results concerning the mediation of subjective norms related to behavior.

**Table 2:** Indirect effects of attitude on behavior through subjective norms and perceived behavioral control

	Point estimate	SE (boot)	Bootstrapping BC 95 % CI	
			Lower	Upper
Visiting STI services regularly				
Subjective norms	−0.00	0.02	−0.04	0.04
Perceived behavioral control	0.08	0.03	0.04	0.15^a^
Adherence to STI treatment				
Subjective norms	0.13	0.11	0.01	0.46^a^
Perceived behavioral control	0.29	0.17	0.10	0.63^a^
Taking an HIV test				
Subjective norms	0.03	0.03	−0.01	0.09
Perceived behavioral control	0.03	0.02	0.01	0.07^a^
Picking up the result of HIV test				
Subjective norms	−0.01	0.02	−0.05	0.02
Perceived behavioral control	0.13	0.04	0.06	0.22^a^

^a^Significant mediation

**Table 3:** Indirect effects of subjective norms on behavior through attitude and perceived behavioral control

	Point estimate	SE (boot)	Bootstrapping BC 95 % CI	
			Lower	Upper
Visiting STI services regularly				
Attitude	0.01	0.03	−0.04	0.07
Perceived behavioral control	0.10	0.04	0.05	0.19^a^
Adherence to STI treatment				
Attitude	0.09	0.19	−0.26	0.51
Perceived behavioral control	0.37	0.21	0.11	0.93^a^
Taking an HIV test				
Attitude	−0.04	0.05	−0.15	0.02
Perceived behavioral control	0.06	0.03	0.02	0.12^a^
Picking up the result of HIV test				
Attitude	−0.01	0.02	−0.06	0.02
Perceived behavioral control	0.12	0.04	0.05	0.21^a^

^a^significant mediation

With regard to the relationships of attitude with behavior (Table 2), the mediation model showed that with regard to all four behaviors, PBC was a mediator. In one of the behaviors, subjective norms was also a mediator.

With regard to the relationships of subjective norms with past behavior (Table 3), the mediation model showed that with regard to all four behaviors, PBC was a mediator.

## Discussion

The aim of this study was to test whether the psycho-social determinants as defined by the TPB provided a suitable explanatory model for visiting STI services, adherence to STI treatment, taking an HIV test and picking up the result of HIV test among *waria*.

In the present study, the TPB variables were related to all four behaviors. The explained variance in behavior (Nagelkerke’s *R*^*2*^) ranged from 15 % up to 70 %. The Nagelkerke *R*^*2*^ is conceptually similar to the *R*^*2*^ in the linear regression. It can vary between 0 (indicating that the predictors are useless in predicting the outcome variable) and 1 (indicating that the model predicts the outcome variable perfectly) [22]. The above findings are in line with reviews on the application of the TPB in the domain of health, for example by Godin and Kok (1996): They show an average explained variance in behavior of 34 % [13]. Armitage and Conner (2001) reviewed 185 studies and found that the TPB accounted for 27 % of the variance in behavior [23]. Thus, we showed that the TPB was able to explain each of the four behaviors to a substantial degree; for the very first time among *waria*.

However, the psycho-social determinants in the TPB variables were differentially related to the four behaviors. Among the three TPB variables, PBC was the component that emerged as the most important and powerful predictor of all four behaviors. This finding was in line with the results of previous meta-analysis that PBC contributes significantly to the prediction of behavior, even after controlling for the effects of the theory of reasoned action variables; attitude and subjective norms [23]. The pattern of results involving PBC was consistent across the health behaviors studied. To summarize, the results of this study complement the findings of previous studies that PBC is the strongest predictor of behaviors across a wide variety of activities including HIV-related health-seeking behaviors.

Regarding attitudes and subjective norms, the logistic regressions suggested that they were less important as predictors: Attitude and subjective norms were not predictive of the four actual behaviors. Therefore, we examined possible mediation. The mediation tests showed that the relationships of attitude with the four behaviors, and the relationships of subjective norms with the four behaviors were all mediated by PBC. This implies that although they had no direct relations with the behaviors, they still are important to address in preventive interventions.

Attitude may become more positive when the PHCs (Primary Health Center/clinics) are improving the quality of services provided such as “One roof services” (STI and HIV test services are available at one place) and “1 day services” (the PHCs/clinics release the result of HIV test at the same day when the patients take an HIV test).

Subjective norms may become more positive when the health providers are able to show friendlier, non-judgmental behaviors and positive (non-)verbal behaviors when they are providing services to the patients. When health providers act in this way, *waria* may recognize the consistency between subjective norms (to always find appropriate medical treatment when they are infected with STIs) and acceptance of health providers to provide the services to this specific group of patients. These kinds of circumstances indirectly will help patients to reduce and overcome unpleasant feelings such as being stigmatized and socially judged for sinful behavior; the task of engaging in the specified behaviors will become easier. The improvement of the quality of the post-test counseling (counseling for the patients after taking an HIV test) is also important. High quality counseling may help patients reduce uncertainty about their future by helping to anticipate and self-regulate the necessary changes in their lives and social relationships.

In conclusion, although subjective norms and attitude had no direct relationships with behavior, they are important for understanding health-seeking behaviors and developing interventions.

It is important to understand the mediated relationships. First, people’s ideas about what others think they should do (subjective norms) may influence their own attitude towards the topic. It may concern a basic mechanism of social influence, in which *waria* adopt others’ opinions to understand reality and adopt seemingly shared values as their own values. Secondly, people’s ideas about what others think they should do (subjective norms) may influence their own perceived behavioral control by modeling. That is, *waria* may observe or hear about others engaging in the tasks of attending STI services regularly and taking an HIV test. This may signal that these behaviors are feasible and socially desirable. Again, the social environment provides basic information on how to regard accessing HIV-related health services.

The present study had some relevant limitations: First, this study was cross-sectional and, therefore, we had to assess the best parameters of future behaviors that were available; past behaviors. That is, it was not feasible to approach the *waria* population twice; once to assess the TPB determinants and later to assess their (future) behavior. We assessed recent (within the past 6 months) behaviors as past behaviors are consistent predictors of future behavior [24–27].

The second limitation is that the survey was conducted by rolling out a structured face-to-face interview, targeting a specific population who were accessing HIV-related health services. HIV-related health services and health-seeking behaviors are commonly associated with sexual risk behaviors and associated with the negative consequences of the risky behaviors. Therefore, the patients and their diseases are very often stigmatized, and it can be expected that respondents may have felt threatened or embarassed when being asked about their personal health issues. This may have stimulated some respondents to provide inaccurate answers. In addition, interviews are more vulnerable to social desirability bias [28], especially because *waria* have been exposed to prevention interventions and know what is “desired”. However, the selection of the interview as a method has been considered carefully. Given that the survey was developed to study health-seeking behavior among a minority and marginalized population (i.e., *waria*), the completeness, truthfulness and accurateness of information collected very much depend, first, on the ability of *waria* to understand the questions. Indeed, the interviewers reported some challenges during data collection regarding *waria’s* limited understanding of the meaning of questions related to sexual and HIV-related health-seeking behaviors. In some cases interviewers needed to use local and slang language, combined with *Bahasa* Indonesia as the common language. In addition, the illiteracy level among *waria* is still very high and, therefore, the respondents were more dependent on the interviewers to respond. The second challenge that supported the decision to use interviews was related to the data being collected in the field at a variety of venues - often in inconvenient settings - making respondents hardly committed to fill in a questionnaire. Considering this situation, the authors preferred to provide full guidance by involving well-trained interviewers rather than letting the respondents fill in the questionnaires independently. The study of Dare and Cleland (1994) shows that in the face-to-face interview, the level of item non-response is low [28].

## Conclusions

Notwithstanding these limitations, the current study has implications for the development of HIV/AIDS prevention interventions by targeting and improving the performance of HIV-related health-seeking behaviors in this particular group. First, this study provides sufficient support for the TPB in explaining HIV-related health-seeking behaviors. Consistent with the formulation of the TPB, PBC significantly predicted behavior. Moreover, it was the strongest predictor. Secondly, this study suggests, despite the fact that PBC is the strongest predictor, prevention interventions should not only focus on strengthening the PBC, but also on shaping positive attitudes and subjective norms among the target audiences. These two predictors seem to have contributed the perceived of control over performance of the four behaviors.

In conclusion, besides educating *waria* about the consequences of not performing the behaviors (attitude), and about how important others find these behaviors (subjective norms), and by providing them with information that makes it easier to engage in the behavior (PBC), care should be taken to provide *waria* with positive experiences with the behavior in the context of the services. Positive experiences may shape positive attitude, a positive subjective norms and it may increase PBC.

## Abbreviations

STIs: Sexually transmitted infections
IBBS: Integrated biological and behavioral survey
MOH RI: Ministry of Health Republic of Indonesia
GFATM: Global Fund to Fight AIDS, Tuberculosis and Malaria
SUM1: Scaling up for most-at-risk populations 1
USAID: United States Agency for International Development
WHO: World Health Organization
TPB: Theory of planned behavior
PBC: Perceived behavioral control
PHC: Primary Health Center
CI: Confidence intervals
IVs: Independent variables
DVs: Dependent variables
